# Valproic Acid-Associated Acute Pancreatitis: Systematic Literature Review

**DOI:** 10.3390/jcm12186044

**Published:** 2023-09-19

**Authors:** Monica C. M. Bischof, Mariana I. E. Stadelmann, Simone Janett, Mario G. Bianchetti, Pietro Camozzi, Barbara Goeggel Simonetti, Sebastiano A. G. Lava, Gregorio P. Milani

**Affiliations:** 1 Family Medicine Institute, Faculty of Biomedical Sciences, Università della Svizzera Italiana, 6900 Lugano, Switzerlandmariana.stadelmann@gmail.com (M.I.E.S.); camozzipietro@gmail.com (P.C.); 2 Department of Pneumology, Ente Ospedaliero Cantonale, 6500 Bellinzona, Switzerland; simone.janett@usz.ch; 3 Sleep Center, Neurocenter of the Southern Switzerland, Ente Ospedaliero Cantonale, 6900 Lugano, Switzerland; 4 Pediatric Institute of Southern Switzerland, Ente Ospedaliero Cantonale, 6500 Bellinzona, Switzerland; barbara.goeggelsimonetti@eoc.ch; 5 Faculty of Biomedical Sciences, Università della Svizzera Italiana, 6900 Lugano, Switzerland; 6 Pediatric Cardiology Unit, Department of Pediatrics, Centre Hospitalier Universitaire Vaudois, University of Lausanne, 1011 Lausanne, Switzerland; webmaster@sebastianolava.ch; 7 Clinical Pharmacology & Therapeutics Group, University College London, London WC1N 3JH, UK; 8 Pediatric Unit, Fondazione IRCCS Ca’ Granda Ospedale Maggiore Policlinico, 20122 Milan, Italy; milani.gregoriop@gmail.com; 9 Department of Clinical Sciences and Community Health, Università degli Studi di Milano, 20122 Milan, Italy

**Keywords:** acute pancreatitis, fatality rate, valproic acid

## Abstract

Long-term medication with valproic acid has been associated with acute pancreatitis. The purpose of this report is to gain insight into the features of this pancreatitis. A preregistered literature search (CRD42023438294) was performed on the National Library of Medicine, Excerpta Medica, Web of Science, and Google Scholar. Patients with alcohol abuse disorder, gallstone disease, hypertriglyceridemia or hypercalcemia, patients with acute valproic acid intoxication, and patients with a pre-existing pancreatitis were excluded. For the final analysis, we retained 73 reports published between 1979 and 2023, which described 125 subjects (83 children and 42 adults predominantly affected by an epilepsy) with an acute pancreatitis related to valproic acid. The diagnosis was made 11 (3.0–24) months (median and interquartile range) after starting valproic acid. One hundred and five cases (84%) recovered and twenty (16%) died. Sex, age, dosage or circulating level of valproic acid, latency time, prevalence of intellectual disability, and antiepileptic co-medication were similar in cases with and without a lethal outcome. Nineteen subjects were rechallenged with valproic acid after recovery: sixteen (84%) cases developed a further episode of pancreatitis. In conclusion, pancreatitis associated with valproic acid presents at any time during treatment and has a high fatality rate.

## 1. Introduction

Acute pancreatitis is an inflammatory process of the pancreas that may occur as an isolated event or recur [1,2]. It is a heterogeneous condition that ranges from minimal inflammation to extensive pancreatic necrosis. Although acute pancreatitis mainly results in exocrine dysfunction, repeated episodes of inflammation and subsequent fibrosis can also result in endocrine insufficiency [1,2]. Chronic alcohol use disorder, gallstone disease, severe hypertriglyceridemia, and hypercalcemia are the most common triggers of acute pancreatitis [1,2]. There is also, but less frequently, an association with genetic factors or with infections of viral (mumps, coxsackievirus, cytomegalovirus, varicella, herpes simplex virus), parasitic (toxoplasma, cryptosporidium, ascaris), fungal (aspergillus), or bacterial (mycoplasma, legionella, leptospira, salmonella) origin [1,3]. Affected patients present with acute-onset severe epigastric and left upper quadrant abdominal pain, usually associated with nausea and vomiting [1].

Finally, there is a link between pancreatitis and drugs [4]. Following the first cases of drug-related pancreatitis reported in the 1950s, about 500 drugs have been temporally associated with pancreatitis [4]. However, most of the evidence comes from individual case reports, which are frequently incomplete, with inadequate data regarding drug dose, and latency between initiating the drug and development of acute pancreatitis, and without exclusion of other common causes.

Valproic acid (dipropylacetic acid), a branched-chain carboxylic acid, is currently recommended to treat epilepsy, acute mania, and bipolar disorders and to prevent migraine headache [5]. While generally considered safe, valproic acid is associated with adverse effects such as skin rashes, bone marrow suppression, liver or kidney injury, hyperammonemia, and teratogenicity [5,6,7].

Valproic acid has also been incriminated as a cause of acute pancreatitis since 1979 [5,6]. However, patients’ age, time latency from initiation of valproic acid to the disease onset, imaging data, and outcome of acute pancreatitis temporally associated with valproic acid are not well characterized because the available literature has not been investigated by means of a structured systematic analysis. The purposes of this review were to illustrate the features of the pancreatitis, to evaluate the predisposing factors, and to speculate on the underlying mechanisms.

## 2. Materials and Methods

### 2.1. Data Sources—Searches

This work was recorded at the International Prospective Register of Systematic Reviews (CRD42023438294) and carried out according to the Joanna Briggs Manual and the second edition of the Preferred Reporting Items for Systematic Reviews and Meta-Analyses (PRISMA) methodology [8]. The National Library of Medicine, Excerpta Medica, and Web of Science were used for a literature search with no date or language limits for the terms (“valproate” OR “valproic acid”) AND (“pancreatitis” OR “pancreatic”). Google Scholar [9], articles already known to the authors, and the bibliography of each identified report were also screened. Cases published uniquely as abstracts were excluded. The searches were undertaken in January 2023 and repeated monthly until 26 June 2023.

### 2.2. Article Selection

Individually documented patients on valproic acid with findings consistent with acute pancreatitis were of interest. In a first round, the results of the initial literature search were screened based on title and abstract. In a second round, the text of the remaining reports was assessed.

### 2.3. Inclusion Criteria

Qualified for the study were individuals on long-term (≥4 weeks) therapy with valproic acid presenting with acute-onset abdominal pain, vomiting or distension and at least one of the following [1,2,3]: 1. amylase or lipase values three times or more the upper limit of normal (the normal values recommended in the literature were taken into consideration for communications, which did not include any reference); 2. imaging studies, intra-surgical or autoptic findings disclosing features consistent with an acute pancreatitis.

### 2.4. Exclusion Criteria

Patients with chronic alcohol abuse disorder, gallstone disease, severe (≥11.4 mmol/L) hypertriglyceridemia or hypercalcemia, patients with acute valproic acid intoxication, and patients with a pre-existing chronic pancreatitis were excluded [1,2]. Subjects with an isolated asymptomatic amylase or lipase elevation were also excluded.

### 2.5. Data Extraction

The following information was collected for each included case, using a predesigned extraction form: 1. sex and age; 2. co-existing chronic kidney disease; 3. underlying cerebral conditions with emphasis on epilepsy, bipolar disorder, and migraine headache; 4. co-existing intellectual disability; 5. antiepileptic co-medication; 6. amylase, lipase or liver enzyme levels, and imaging studies, intra-surgical or autoptic findings; 7. latency from initiation of valproic acid to the development of pancreatitis, dosage, and circulating level of valproic acid; 8. clinical course; and 9. occurrence of recurrences after reintroducing valproic acid.

### 2.6. Comprehensiveness of Reporting

The nine data extracted from each individual pancreatitis case were rated as 0 or 1, and the reporting comprehensiveness was graded according to the sum as excellent (≥7), good (5 to 6), or satisfactory (4 to 5).

### 2.7. Analysis

Two authors in duplicate but not independently conducted the literature search, the selection of eligible studies, the data extraction, and the assessment of the comprehensiveness of each included case. In the event of any disagreements, a discussion took place to resolve them, with the participation of a senior author to address any remaining discrepancies. One author entered the data into a pre-defined worksheet, and the second author verified the accuracy of the data entry.

To address missing data, pairwise deletion was employed [10]. Categorical data are presented as counts. Fisher’s exact test was utilized for dichotomous data, while the unpaired two-samples Mann-Wilcoxon test was employed for ordered categorical data [11,12]. Continuous data are presented as medians and interquartile ranges, and their analysis was conducted using the Mann-Wilcoxon test [12]. Two-sided *p* values of <0.05 were considered significant. The GraphPad Prism 10.0.0 (GraphPad Software, San Diego, CA, USA) was used for statistics.

## 3. Results

### 3.1. Search Output

**Figure 1:**
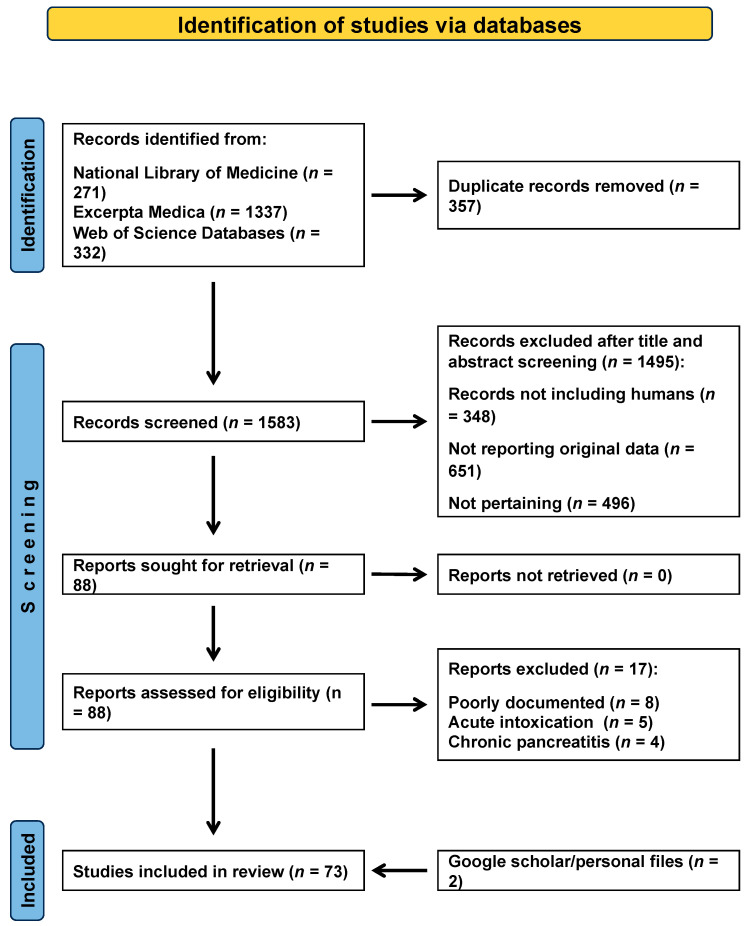
Valproic acid-associated acute pancreatitis—literature search process.

The literature search process is outlined in Figure 1.

For the final analysis, we retained 73 articles [13,14,15,16,17,18,19,20,21,22,23,24,25,26,27,28,29,30,31,32,33,34,35,36,37,38,39,40,41,42,43,44,45,46,47,48,49,50,51,52,53,54,55,56,57,58,59,60,61,62,63,64,65,66,67,68,69,70,71,72,73,74,75,76,77,78,79,80,81,82,83,84,85] published between 1979 and 2023: 28 from America (United States of America, N = 19; Canada, N = 3; Brazil, N = 2; Chile, N = 1; Colombia, N = 1; Peru, N = 1; Venezuela, N = 1), 23 from Europe (France, N = 5; Germany, N = 4; Italy, N = 3; Spain, N = 3; Denmark, N = 1; Estonia, N = 1; Greece, N = 1; Netherlands, N = 1; Poland, N = 1; United Kingdom, N = 1; Switzerland, N = 1; Serbia, N = 1), 18 from Asia (India, N = 6; Japan, N = 5; People’s Republic of China, N = 2; Saudi Arabia, N = 2; Türkiye, N = 2; Malaysia, N = 1), 3 from Oceania (Australia, N = 3), and 1 from Africa (Tunisia, N = 1). Fifty-nine articles were written in English, five in French, three in Spanish, two in German, and one each in Danish, Italian, Portuguese, and Serbian.

The mentioned 73 articles [13,14,15,16,17,18,19,20,21,22,23,24,25,26,27,28,29,30,31,32,33,34,35,36,37,38,39,40,41,42,43,44,45,46,47,48,49,50,51,52,53,54,55,56,57,58,59,60,61,62,63,64,65,66,67,68,69,70,71,72,73,74,75,76,77,78,79,80,81,82,83,84,85] described 125 subjects with an acute pancreatitis temporally related to medication with valproic acid. Reporting completeness was excellent in 46 (37%), good in 52 (42%), and satisfactory in the remaining 27 (22%) cases.

### 3.2. Findings

The characteristics of the 125 patients, 83 children and 42 adults predominantly affected by an epilepsy (93%), are depicted in Table 1.

The diagnosis of acute pancreatitis was made on average 11 months after starting valproic acid: it was occasionally less than 3 months, mostly 3 to 24 months, and occasionally 25 months or more. The valproic acid dosage was less than 50 mg/kg daily in about 80% of cases. A pre-existing chronic kidney disease and signs of a liver involvement were disclosed in about every tenth case.

The disease course was generally rather poorly documented (mainly in patients with a lethal outcome). One hundred and five cases (84%) recovered from the pancreatitis. The recovery time, reported for 44 cases, was less than 2 weeks after diagnosis in 20 (45%), 2–4 weeks in 16 (36%), and 5 weeks or more in 8 (18%) cases. Twenty cases (16%) died in direct relation to the pancreatic disease on average seven days after pancreatitis diagnosis. Sex, age, dosage or circulating level of valproic acid, latency time, prevalence of intellectual disability, and antiepileptic co-medication were not different in patients with and without lethal outcome.

**Figure 2:**
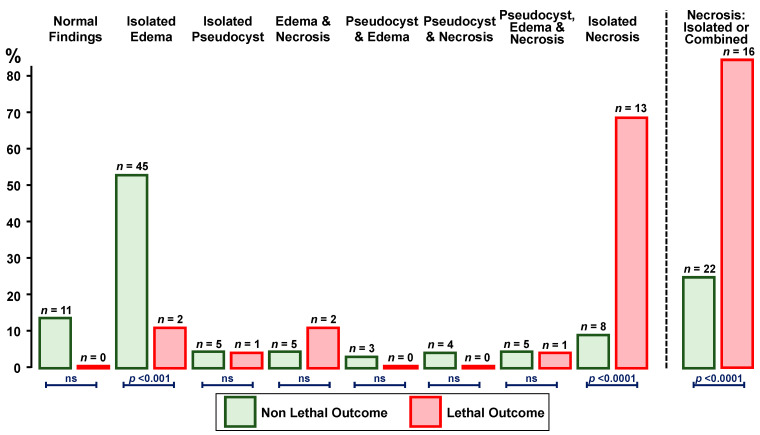
Imaging findings in 105 patients with valproic acid-associated acute pancreatitis (86 without and 19 with lethal outcome). ns = non-significant.

The prevalence of imaging studies disclosing necrosis was significantly higher (*p* < 0.0001) in cases with (84%) than in those without (26%) a lethal outcome (Figure 2).

Nineteen subjects were rechallenged with valproic acid after recovering from pancreatitis. Sixteen (84%) cases developed a second (N = 15) episode, or even more (N = 1), of pancreatitis after re-exposure to valproic acid. In the remaining three (16%) cases, re-exposure to valproic acid was not followed by a pancreatitis recurrence. Finally, two patients had a further episode of pancreatitis without any re-exposure to valproic acid.

## 4. Discussion

This review of the literature documents 125 cases of acute pancreatitis occurring on medication with valproic acid but in absence of other known potential causes of acute pancreatitis (like chronic alcohol abuse disorder, gallstone disease, severe hypertriglyceridemia, or hypercalcemia) [1,2]. The results may be summarized in four points: 1. About two thirds of cases occur in subjects 16 years or less of age. 2. The duration of therapy before pancreatitis varies between weeks and years. 3. There is a fatality rate of approximately 15% (mostly in cases with a necrotizing pancreatitis). 4. Cases without and with a fatal outcome do not differ with respect to age, sex, latency time, dosage, and circulating level of valproic acid.

The failure to demonstrate the most recognized triggers of acute pancreatitis [1,2] and the recurrence of pancreatitis after re-exposure to valproic acid in more than 80% of cases may support the existence of a cause–effect relationship between valproic acid and acute pancreatitis.

In subjects who do not experience abdominal pain, distension, or vomiting, regular pancreatic ultrasounds or monitoring of pancreatic enzymes is of no value for the early discovery of drug-induced acute pancreatitis including valproic acid-associated pancreatitis, mainly because this medication is often accompanied by a transient but not clinically relevant elevation of these enzymes [4]. Clinicians should instead be alert whenever a subject on valproic acid presents with acute onset of abdominal pain, distension, or vomiting. It is true, however, that in subjects with intellectual disability the evaluation of acute abdominal pain is more difficult due to communication barriers and cognitive limitations.

The mechanisms through which valproic acid may induce pancreatitis are elusive. Therapy with valproic acid might result in a mitochondrial injury with subsequent liver or kidney tubular dysfunction [6,87,88]. However, it is currently believed that valproic acid-associated pancreatic injury does not result from a mitochondrial dysfunction but from an accumulation of free radicals secondary to radical scavenger depletion [6,87,88,89].

Valproic acid may also induce a latent chronic pancreatitis, which is detected if it results in an acute-onset symptomatic pancreatitis episode [5,6]. Finally, an acute pancreatic damage, mostly associated with signs of septic shock, such as low blood pressure, high heart rate, and increased body temperature, in addition to respiratory depression, nausea, vomiting, diarrhea, miosis, agitation, trembling, and myoclonus, has been noted in the context of an acute valproic acid poisoning [90].

Since 2000, the term review was used in five reports addressing the development of acute pancreatitis in subjects on therapy with valproic acid [52,53,61,89,91]. However, none of these reports, which included between 33 and 73 cases each, was undertaken by means of a well-structured search process and strategy. Furthermore, some reports also included cases with a pre-existing chronic pancreatic disease and cases with acute overdose, or failed to exclude cases concurrently presenting with widely accepted triggers of acute pancreatitis. Therefore, the current study is the first to systematically assess all the available literature on this issue. Nevertheless, this analysis also has some limitations. First, we did not incorporate several cases (N = 8) which had been insufficiently documented. Second, the valproic acid level was often not reported and information on the disease course was often scanty. Third, we could not analyze the time to diagnosis. Finally, the diagnostic attitude strongly varied between 1979 and 2023. For example, in many cases published before 1990, the diagnosis of acute pancreatitis relied exclusively on amylase but without the lipase level and imaging studies.

## 5. Conclusions

This review outlines characteristics and predisposing factors of valproic acid-associated acute pancreatitis. This condition can present at any time during treatment, should be suspected whenever a subject receiving this agent presents with vomiting and abdominal pain, or distension, and likely has a higher fatality rate than pancreatitis of other causes. Prompt discontinuation of valproic acid and supportive care are the mainstay of therapy. A patient with valproic acid-related acute pancreatitis should not be rechallenged, because the recurrence rate is high. It is imperative to educate caregivers and patients on the presentation of valproic acid-associated acute pancreatitis, but regular enzyme monitoring is unnecessary.

## Figures and Tables

**Table 1:** Baseline characteristics of 125 patients 1.0 to 66 years of age with an acute pancreatitis temporally associated with valproic acid treatment. Results are given as frequency (and percentage) or as median (and interquartile range). N.A. = not applicable, ns = non-significant.

		Outcome		
	All Patients	Non-Lethal	Lethal	*p*-Value
				
*n* (%)	125	105 (84%)	20 (15%)	N.A.
Males, *n* (%)	83 (66%)	72 (69%)	11 (55%)	ns
Age				
years	13 (7.0–21)	12 (7.0–19)	14 (7.1–28)	ns
≤16 years, *n* (%)	83 (66)	70 (67)	13 (65)	ns
				
Underlying condition, *n*	121	102	19	ns
Epilepsy, *n* (%)	112 (93)	94 (92)	18 (95)	ns
Bipolar disorder, *n* (%)	7 (5.7)	6 (5.8)	1 (5.3)	ns
Migraine headache, *n* (%)	2 (1.7)	2 (2.0)	0	ns
Intellectual disability, *n* (%)	49 (39)	42 (40)	7 (35)	ns
				
Further antiepileptic co-medication, *n* (%)	54 (43)	43 (41)	11 (55)	ns
Co-existing chronic kidney disease, *n* (%)	14 (11)	10 (9.5)	4 (20)	ns
				
Duration of treatment with valproic acid, *n*	99	80	19	
months	11 (3.0–24)	11 (3.5–28)	11 (2.9–21)	ns
4–8 weeks, *n* (%)	6 (6.1)	4 (5.0)	2 (11)	ns
9–12 weeks, *n* (%)	22 (22)	17 (21)	5 (26)	
>3–12 months, *n* (%)	28 (28)	24 (30)	4 (21)	
>12–24 months, *n* (%)	18 (18)	14 (18)	4 (21)	
>24 months, *n* (%)	25 (25)	21 (26)	4 (21)	
				
Valproic acid dosage ^#^, *n*	87	73	14	
mg/kg daily	30 (21–45)	30 (20–43)	40 (30–48)	ns
≥50 mg/kg daily, *n* (%)	15 (17)	12 (16)	3 (21)	ns
				
Valproic acid blood level ^∆^, *n*	71	61	10	
µmol/L	520 (411–593)	520 (436–598)	420 (245–531)	ns
≥900 µmol/L, *n* (%)	2 (2.8)	2 (3.3)	0	ns
				
Pathologically altered liver enzymes *, *n* (%)	14 (11)	10 (9.5)	4 (20)	ns
Recurrent valproic acid-associated pancreatitis, *n* (%)	16 (13)	14 (16)	2 (11)	ns

^#^ The typical oral dosage of valproic acid for epilepsy is 10–60 mg/kg/day, most frequently administered in two divided doses [86]. ^∆^ The typical therapeutic range for valproic acid is usually <800 µmol/L, while risk of toxicity starts when it is >900 µmol/L [86]. * Alanine or aspartate aminotransferase two times or more the upper limit of normal.

## Data Availability

The data examined or generated during this study are included in this article and its references. For additional inquiries, interested parties are encouraged to contact the corresponding author.
